# Vibrational imaging of metabolites for improved microbial cell strains

**DOI:** 10.1117/1.JBO.29.S2.S22711

**Published:** 2024-07-01

**Authors:** Adam Hanninen

**Affiliations:** Trestle Optics, Irvine, California, United States

**Keywords:** metabolomics, biomanufacturing, microscopy, cell sorting, infrared, Raman, spectroscopy

## Abstract

**Significance:**

Biomanufacturing utilizes modified microbial systems to sustainably produce commercially important biomolecules for use in agricultural, energy, food, material, and pharmaceutical industries. However, technological challenges related to non-destructive and high-throughput metabolite screening need to be addressed to fully unlock the potential of synthetic biology and sustainable biomanufacturing.

**Aim:**

This perspective outlines current analytical screening tools used in industrial cell strain development programs and introduces label-free vibrational spectro-microscopy as an alternative contrast mechanism.

**Approach:**

We provide an overview of the analytical instrumentation currently used in the “test” portion of the design, build, test, and learn cycle of synthetic biology. We then highlight recent progress in Raman scattering and infrared absorption imaging techniques, which have enabled improved molecular specificity and sensitivity.

**Results:**

Recent developments in high-resolution chemical imaging methods allow for greater throughput without compromising the image contrast. We provide a roadmap of future work needed to support integration with microfluidics for rapid screening at the single-cell level.

**Conclusions:**

Quantifying the net expression of metabolites allows for the identification of cells with metabolic pathways that result in increased biomolecule production, which is essential for improving the yield and reducing the cost of industrial biomanufacturing. Technological advancements in vibrational microscopy instrumentation will greatly benefit biofoundries as a complementary approach for non-destructive cell screening.

## 1 Introduction

The motivation to develop sustainable, carbon-neutral manufacturing practices by leveraging bio-based resources is strong. Since the Industrial Revolution, the global economy has relied on the overexploitation of natural resources and ecosystems, producing increased emissions from anthropogenic sources of greenhouse gases. Strategies to mitigate continued emissions rely on adopting upstream “supply side” solutions for sourcing chemical inputs that prioritize sustainability.^1^^,^^2^ Thankfully, biological-based enzymatically catalyzed processes can efficiently synthesize a plethora of useful metabolic biomolecules without contributing significantly to carbon emissions. These efforts began in a commercial setting in 1978 with the release of *Escherichia coli*-sourced human insulin by Genentech. Now, microbes are used to produce fertilizers, plastic precursors, fragrances, dyes, and nutraceuticals, to name a few.^3^ However, to scale economically viable biomanufacturing beyond high-margin, low-volume products, improved yields from the biological machines, or cell factories, used to produce these biomolecules is necessary.^4^^,^^5^

**Fig. 1:**
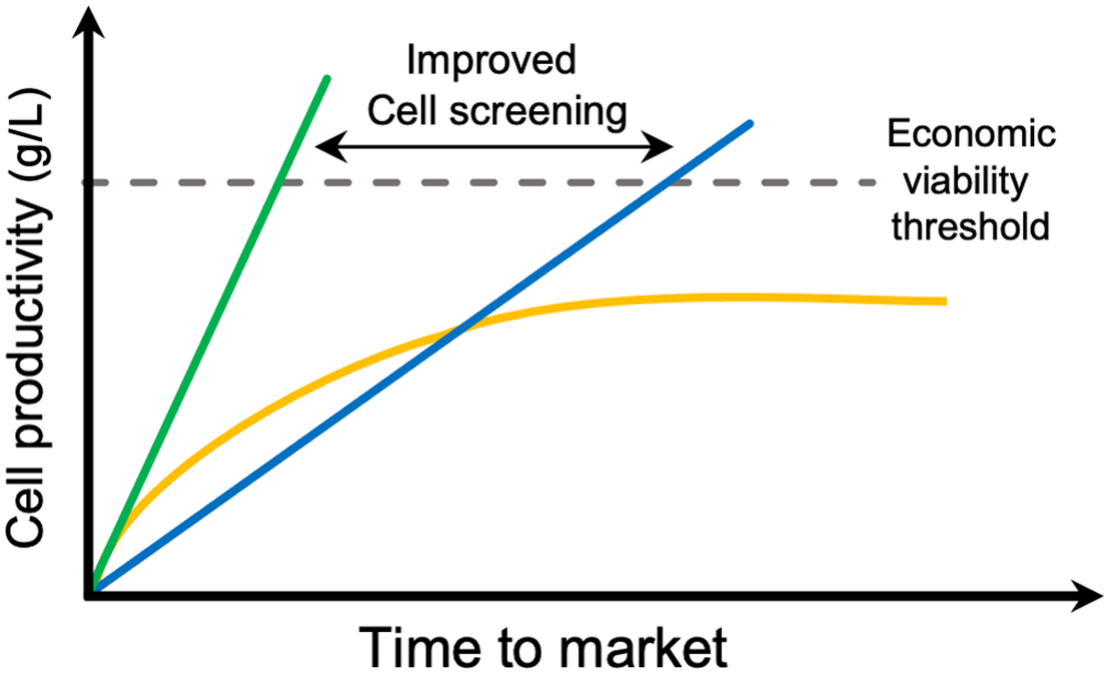
Although not all cell strain development efforts are successful (yellow), with improved cell screening workflows, the time to market can be reduced.

Industrial synthetic biology efforts exploit the advances in genome editing in combination with automation, analytical measurements, and data integration to build high-throughput strain engineering pipelines, also known as biofoundries.^6^^–^^8^ Such biofoundries have optimized laboratory workflows aimed at creating microbial cell factories that produce biomolecules of interest at the lowest cost and in the shortest amount of time possible. These value-added biomolecules, also known as secondary metabolites, and their precursors compose the metabolome and include intracellular as well as excreted biomolecules. The aim of cell development programs is to create strains that produce enough metabolite yield to exceed the economic viability threshold (EVT) (Fig. 1). This threshold determines whether a commercial venture based on a particular cell strain will be sustainable, as measured by profit, which depends on the market dynamics and opportunity for the targeted metabolite. By far, the highest-volume and lowest-margin application for engineered metabolism is the production of transportation fuels. This production represents a very high EVT. Conversely, low-volume and high-margin metabolites such as nutraceuticals, flavorings, and dyes have a lower EVT and tend to be commercialized earlier than more economically challenging applications.

The metabolome is dynamic and reflects the expression of the genome under specific environmental conditions. Interrogating the relative abundance of these metabolites not only provides a snapshot of the metabolic phenotype but also offers insights into how even small metabolic pathway perturbations can lead to major metabolic changes. This has implications for discovering new metabolic pathways that correlate with the regulated activation of specific biosynthetic gene clusters (BGCs).^9^ Connecting BGCs to the metabolites that they encode is a major ongoing challenge mainly because of the presence of pleiotropic genes, which influence multiple, seemingly unrelated phenotypic traits, and silenced BGCs that are not expressed under certain environmental conditions. Deciphering the connection among the genome, the environment, and the resulting metabolome offers the opportunity to enhance the current understanding of biological functions and elucidate their underlying mechanisms, so they may be controlled through metabolic engineering.

The genome, transcriptome, proteome, and metabolome form different layers of the so-called multiomics cascade, each of which characterizes a biosystem or an organism at a different biomolecular layer. Integrative analysis of multiomic datasets using a variety of analytical instrumentation paired with an arsenal of mathematical and computational techniques has helped discover how upstream perturbations influence subsequent layers in the cascade.^10^ Despite recent successes, the extreme challenge of relating genotype to phenotype remains the central dogma of molecular biology. Although targeted gene mutagenesis techniques including the indispensable CRISPR/Cas9 allow for the creation of libraries of genetic variants, these targeted techniques are often paired with random mutagenesis (e.g., ultraviolet or chemical exposure) to further increase genetic variation with the hope of improving the phenotypic traits of the organism.^11^^–^^13^ The rate at which genetic libraries are easily generated, however, vastly dwarfs the rate at which these genetic variants are tested or screened due to the limited throughput of existing analytical instrumentation. This difference is further compounded once dynamic changes in environmental factors are considered, especially once multiple microbial species are introduced.^14^ One of the more potent strategies to induce silenced pathways and enhance the chemodiversity of microorganisms is through microbial co-cultivation, which mimics the competitive environment in unmanaged, natural environments, e.g., the soil rhizosphere and aquatic phycosphere, thus further expanding the variability in possible microbe phenotype.^15^^,^^16^ The severe mismatch between the relative ease of building and cultivating a diverse set of cell variants, versus the tedious process of measuring the metabolite content, as part of the design, build, test, and learn cycle, emphasizes the clear need for improved high-throughput screening technologies. Equally critical is the ability to preserve cell integrity for selective propagation or for the corresponding genetic information and BGCs to be identified through follow-on gene sequencing assays, enabling a more meaningful and comprehensive analysis of the sample. In other words, if a cell is found to be a high producer of a particular metabolite, it is of the utmost importance that the cell survives for both propagation and additional analysis. Unifying these attributes into a quantitative analytical platform will greatly expedite the microbial cell factory development pipeline for biomanufacturing applications.

Industrial microbial cultivators typically deploy axenic bacteria or yeast cultures grown in environmentally controlled bioreactors, or microalgae species, notably *Chlorella* and *Spirulina*, cultivated in open raceway ponds and closed photobioreactors.^17^ Prior to at-scale production, cell strain development programs use analytical technologies, including mass spectrometry (MS) coupled with liquid and gas chromatography, nuclear magnetic resonance (NMR) spectroscopy, and fluorescence during metabolite characterization studies.^18^ Despite their successes, new tools are needed to facilitate the non-invasive acquisition of molecular-specific information that can be used to guide researchers in the design and build stages of strain engineering.^19^ Novel analytical techniques in vibrational spectro-microscopy based on Raman scattering and infrared (IR) absorption contrasts are now being developed and hold great potential for non-invasive high-throughput cell screening. The utility of these nascent imaging methods for synthetic biology is realized when integrated with microfluidic systems equipped with real-time image processing and automated cell sorting.

## 2 Methods for Metabolomics

### 2.1 Mass Spectrometry

MS remains the dominant technology used for the quantification of natural product targets and is the most widely used platform for profiling engineered biosynthetic pathways in the microbial metabolome.^20^ MS and its many variations benefit from their ability to quantitatively detect a diverse class of metabolites with both a low chemical concentration limit of detection (LOD) and over a large concentration range. The challenge for this class of analytical technologies is to achieve the screening throughput required to meet the needs of biofoundries. In addition, persistent difficulties in confidently identifying compounds during fragment annotation of untargeted metabolites severely limit the utility of the MS dataset. MS can be deployed for high-resolution and even three-dimensional (3D) imaging using approaches such as secondary ion MS, and sensitivity can reach the single molecule limit.^21^ Although MS provides a snapshot of the microbial molecular composition, it has a significant drawback in that the cells are lysed during the ionization process required to measure metabolite content. Consequently, all spatial, morphological, and dynamic information of the cell is lost, and it excludes the ability to perform follow-on multiomic assays, including gene sequencing. Furthermore, it eliminates the possibility for researchers to save high-producing cells for the selective propagation needed to realize enriched cell strains through directed evolution, which is the *raison d’être* of synthetic biology.

### 2.2 Nuclear Magnetic Resonance

NMR spectroscopy offers several unique advantages over other metabolomic platforms in that it is non-destructive and quantitative, requires little sample preparation, and is amenable to detecting hard-to-ionize compounds that can be challenging to detect through MS.^22^^,^^23^ Because the tool is non-destructive, NMR is suitable for tracking metabolic flux in living cells across longitudinal studies. Under ideal automation circumstances, an NMR system can collect and process $∼10^{2}$ to $10^{3}$ samples per day, similar to MS.^24^ Although the chemical concentration limits of detection are typically ∼1  μM, the technique can achieve a linear concentration response in the 10  μM to 1 M range.^25^ In addition to throughput, the primary obstacle to the routine application of NMR for metabolomics is the poor voxel spatial resolution of several microns, comparable in size to most microbe species preventing sub-cellular resolution, and high levels of signal overlap among different metabolites. The latter can be deconvoluted using two-dimensional (2D) NMR methods at the expense of throughput as these are considerably more time consuming and computationally intensive. Alternatively, stable isotope probes with a nonzero nuclear spin (e.g., C13, N15, and P31) can be used to identify biomolecules of interest by separating their signals from the background compounds. A potent advantage of H1 proton NMR is the ability to provide information about chemical shifts by probing the proton’s localization to electronegative elements or groups.^26^ This provides unique information on the molecular structure, conformation, and composition, as well as detail on 3D protein folding. Although the approach is generally slow, integrating NMR with other analytical tools with high throughput can provide highly useful confirmatory and complementary information on the underlying presence and structure of metabolites in living cells.

### 2.3 Fluorescence

Fluorescence is a diverse field, encompassing many techniques that have become ubiquitous throughout microbiology and the life sciences to non-destructively track morphological and functional information within cells. When deployed in a microscope, fluorescent dyes can rapidly localize biomolecules with subcellular resolution and can reach single-molecule sensitivity. Although some biomolecules have intrinsic autofluorescence properties, most do not, and therefore, measurements instead rely on exogenous fluorescent probes to identify chemical species such as proteins, hormones, metabolites, and more through immunofluorescence.^18^ Alternatively, genetically encoded fluorescent reporters, such as green fluorescent protein and its numerous derivatives, are encoded through modified DNA introduced to the cell and routinely used in cell strain engineering to provide a minimally perturbative window into the real-time inner workings of the biochemistry in living cells. Combining these techniques with vibrationally encoded^27^ or fluorescence lifetime imaging^28^ and super-resolution microscopy^29^ should allow for simultaneous, highly multiplexed visualization and quantification of many chemical targets with localization beyond the diffraction limit.

Compared with conventional fluorescence microscopy paired with microtiter plates and manual pipetting, innovations in fluorescence used with flow cytometry have dramatically accelerated the speed of cell screening, reaching up to $10^{3}$ to $10^{4}$ cells per second.^30^ Combining the approach with real-time signal processing and cell sorting, sometimes known as fluorescence-activated cell sorting, allows cells with desirable metabolite features to be identified and sorted for follow-on assays or selective propagation.^31^ This automates the decision-making process and significantly helps researchers simplify their discovery-making workflow. Flow cytometry relies on an illumination source, typically a laser, to illuminate cells as they flow through the interrogation region of the microfluidic channel. If fluorescent probes have accumulated in the cell, the laser will excite the fluorophores, resulting in an intensity-based fluorescence signal captured by a photodetector. Given the speed of flow cytometry systems, implementations typically rely on fluorescence spectroscopy as morphological information is not captured, although image-based cell sorting instruments with high frame rate imaging detectors are available.^32^ A limitation of the approach is that the fluorescence intensity does not always correspond to increased metabolite production, especially with intracellular metabolites, as increased accumulation has been shown to result from competing factors, including the fluorophore’s permeability through the cell membrane.^33^ Moreover, fluorescence flow cytometry is susceptible to the longstanding issues related to fluorescence. Exogenous probes are known to perturb native cellular functions, especially when used to label small molecules, and strong light dosages can cause cell lysis from phototoxicity. Fluorophores can also photobleach, rendering them incapable of emitting fluorescent radiation. This limits their ability to track metabolites in longitudinal studies, for example, across the growth stage of cells or over multiple generations of cell production. Given these limitations, there is a need for label-free imaging methods to rapidly screen cells with endogenous molecular contrast.

## 3 Vibrational Imaging

The advent of novel vibrational spectro-microscopy methods with increased sensitivity and signal-to-noise ratio (SNR) has enabled real-time structural and functional measurements of single cells. These innovations provide intrinsic chemical contrast by probing spectroscopic signatures, which serve as a molecular “fingerprint” to differentiate cell species and phenotype without prior knowledge of the cells.^34^ Aspects of an ideal spectroscopic imaging technique would provide biologically relevant chemical and morphological information about intracellular activity including the cascade of signal transduction events as well as intercellular chemical signaling including metabolite exudates while remaining label-free as to not perturb the native biology and in real time. A central challenge of vibrational imaging methods is the ability to detect molecularly similar but distinct chemical species with specificity. Another challenge relates to the detection of low-abundance chemical species that may be present at nanomolar concentrations without the aid of fluorescence labeling. Methods aimed at addressing these limitations must further consider measurement throughput, which can impose practical limits on the ability to screen large populations of cells.

**Fig. 2:**
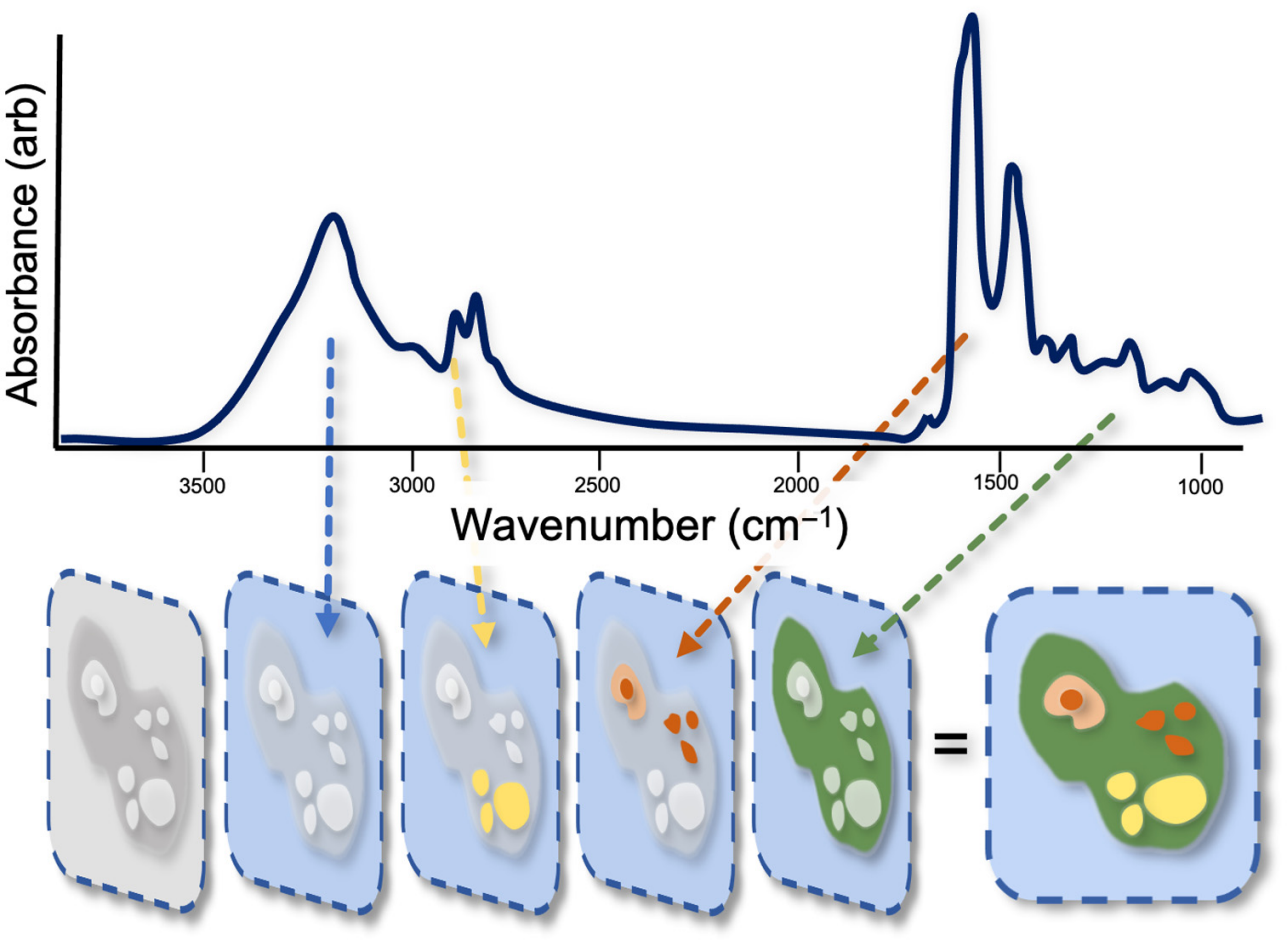
Instead of capturing gray-scale images, through hyperspectral imaging, quantitative maps of chemically distinct structures can be visualized. This “adds color” to the images and can be achieved through both IR- and Raman-based imaging.

There are two general approaches to exciting vibrational resonances, Raman scattering and IR absorption. Both spectroscopic techniques are powerful optical-based approaches used for label-free chemical mapping and have found their way into laboratories in academic, industrial, and clinical settings, where they are used to image a wide variety of specimens, including sectioned tissue samples, engineered cell cultures, and pharmaceutical products.^35^^–^^37^ They provide direct measurements of the chemical landscape for cellular phenotyping, which can be used to identify cells with a high metabolite yield. Provided that the chemical contrast is based on molecular vibrational resonances, these approaches are especially useful for identifying molecular species found in biology composed of elements, including carbon, hydrogen, nitrogen, oxygen, phosphorus, and sulfur. Between these complimentary spectroscopic approaches, Raman-based techniques are currently more technologically sophisticated due, in part, to advancements in tunable ultrafast light sources, high-sensitivity silicon detectors, and compatibility with existing visible to near-IR optical microscope instruments. Nevertheless, IR absorption is a much stronger light–matter interaction, and developments are underway to facilitate the integration of IR-based methods with standard optical microscopes (Fig. 2).

### 3.1 Raman Spectro-Microscopy

Raman scattering encompasses a family of imaging techniques that indirectly probe nuclear motions in a molecule through the motion of electrons, known as electronic polarizability. Of particular interest are stimulated Raman scattering (SRS)-based methods, which have shown enormous potential for rapid molecular mapping. SRS is a coherent process that excites ensembles of molecules through constructive phase-matched interference, yielding signals that can be orders of magnitude stronger than linear Raman signals. SRS and coherent anti-Stokes Raman scattering (CARS) are successful laser-scanning imaging implementations capable of probing microscopic volumes with pixel dwell times of microseconds or less.^38^^–^^40^ Improvements in laser performance and signal detection techniques have helped improve chemical concentration sensitivity, but they do not fundamentally overcome the low Raman cross-sections of many molecular systems. Some Raman-active chemical markers that have become mainstays for SRS imaging include the $3015\;{\mathrm{cm}}^{-1}$ band associated with the C=C–H stretching modes in unsaturated fatty acids and the ${\mathrm{CH}}_{2}/{\mathrm{CH}}_{3}$ modes around 2845 and $2950\;{\mathrm{cm}}^{-1}$, respectively.^41^^–^^43^

**Fig. 3:**
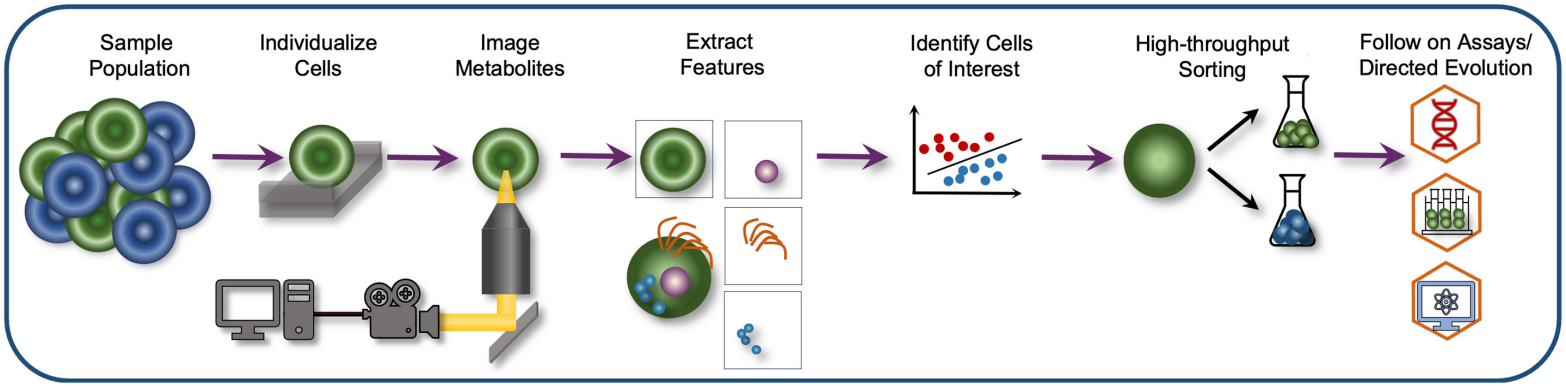
Workflow for image-based metabolite screening. Cells from within a population are individualized in a narrow microfluidic channel where they are then interrogated using an optical microscope. The images are rapidly analyzed, and cells of interest are saved for either additional follow-on assays, including MS/gene sequencing, or for directed evolution.

Recent innovations have pushed the approach to even greater throughput by combining an SRS microscope with microfluidic devices to rapidly characterize the metabolite content of cells in a flow channel.^44^^,^^45^ One such high-throughput development, known as Raman image-activated cell sorting (RIACS),^46^ provides real-time image acquisition and image analysis to enable automated decision-making regarding whether the interrogated cell should be sorted into the collection channel or the waste channel. This approach uses an SRS microscope and deploys a field-programmable-gate-array–computer processing unit infrastructure for rapid image processing with fluidic and mechanical devices capable of real-time and non-perturbative sorting of up to ∼100  events/s. Although SRS is typically performed in a point-scanning geometry using galvanometric mirrors, the RIACS setup uses a line-focused pump and Stokes beams in the direction perpendicular to the flow of the fluidic channel to acquire relatively blur-free multiplexed images. Pairing this hardware with sophisticated machine learning (ML) analytics will further enhance the utility of the approach (Fig. 3).

RIACS holds promise to deliver fast, label-free chemical imaging suitable for strain engineering applications, including separating microbial species grown in co-culture settings or for sorting high producer cells from within a population. The SRS mechanism, however, has a lower chemical concentration LOD of ∼1  mM for many molecular species, rendering the approach incapable of detecting biomolecules produced in lower concentrations. Electronic excitations can enhance the Raman response and thus boost the sensitivity of the measurement, but for most biomolecules, this is in the harsh ultraviolet regime and would compromise cell integrity.^47^ Recent innovations based on stimulated Raman photothermal (SRP)^48^ contrast have shown improved sensitivity in the sub-millimeter regime by probing the transient thermal lensing effect caused by a phonon-mediated relaxation from the excited vibrational state using a third pulsed light source, i.e., the probe beam. This will be useful for detecting chemical species that have low concentrations or relatively low Raman activity and may offer a path toward improved cell screening throughput without compromising the SNR.

### 3.2 IR Spectro-Microscopy

IR absorption spectroscopy is a popular technique for detecting and analyzing the chemical composition of samples. When incorporated in a microscope, the IR spectroscopy technique can be used to produce images with genuine spectroscopic contrast, allowing for non-destructive and non-invasive label-free imaging.^49^^–^^52^ IR absorption is complementary to Raman scattering as it probes IR-active molecules with permanent dipole moments through a direct dipole-allowed transition to an excited vibrational state. Of the two types of vibrational light–matter interactions, the IR-induced transition using mid-IR (MIR) radiation (λ=3 to 10  μm) is by far the strongest, characterized by IR absorption cross-sections that are $∼10^{8}$ greater than the corresponding Raman scattering cross-section. ^53^ This virtue allows IR-based methods to be deployed in a widefield format as opposed to Raman-based methods, including SRS and CARS, which require tight optical focusing to acquire images. Widefield imaging using cameras can increase the imaging speed by ∼100× without a loss in the SNR, allowing for a dramatic enhancement in imaging throughput over beam-scanning methods.

As an analytical technique, IR imaging has had a significant impact on a wide range of commercial and biologically relevant samples. Despite the proven successes of IR imaging, the approach suffers from practical hurdles restricting its utility; in particular, IR imaging exhibits a much lower spatial resolution compared with visible light microscopy due to the long wavelengths used to resonantly excite nuclear vibrational motion. Progress in MIR photonic tools such as tunable quantum cascade lasers is being made by multiple suppliers; however, there are limited commercial offerings for IR-compatible optical components compared with the visible and near-IR, including objective lenses, and IR cameras suffer from thermal noise, generally feature low pixel densities, and are much less affordable compared with Si-based detectors.^54^^,^^55^ These detriments have prevented greater adoption of IR imaging techniques used to study samples at the single-cell level.

Fortunately, recent innovations in nonlinear IR spectro-microscopy are poised to overcome spatiotemporal limitations and have laid the foundation for a revolution in rapid IR imaging at high speed and sub-cellular resolution. Third-order sum-frequency generation (TSFG)^56^^–^^58^ and photothermal-IR (PI) microscopy^59^^–^^61^ have broken the IR-diffraction limit and provided sub-micron resolution over the entire MIR spectral range by encoding the long wavelength IR photons used to excite vibrational resonances onto short wavelength visible photons. These techniques use narrowband, tunable MIR light sources to excite molecular vibrational motions, which are in turn detected with the aid of high-performance silicon-based photodetectors, a feature that is highly attractive for biological imaging studies on standard visible microscopes.

In TSFG, a MIR-driven vibrational coherence is probed with a two-photon up-conversion (hyper-Raman) interaction, producing a coherent signal in the visible part of the spectrum. The four-wave mixing technique can be incorporated into a laser-scanning microscope, offering straightforward 3D visualization of biological samples in a manner similar to SRS and CARS but with the chemical contrast based on the IR activity of molecular vibrations. Although efforts to date have focused on vibrational imaging in the C–H range (∼2800 to $3000\;{\mathrm{cm}}^{-1}$), the real benefits of TSFG are likely to be found in the fingerprint region (∼800 to $1800\;{\mathrm{cm}}^{-1}$) where several biomolecules including proteins express prominent IR-active vibrational modes as well as in highly conjugated molecular systems. To facilitate collinear illumination and tight focusing over an extended spectral range from the visible/near-IR through the entire MIR, optical components with very broad spectral transmission and limited chromaticity, such as reflective components, are required. Reflective Schwarzschild–Cassegrain objectives have poor focal volume confinement compared with similar refractive objectives due to the center obscuration caused by the smaller convex mirror; however, innovations in freeform optical design of reflective objectives are underway to remove the obscuration and provide diffraction-limited performance, which will likely alleviate these drawbacks and unlock the full potential of the TSFG modality for label-free, high-resolution vibrational imaging.

Another technique that has garnered significant attention is PI microscopy. Unlike TSFG, PI microscopy is not a coherent process, relaxing the requirement for collinear illumination. This instead allows oblique and counter-propagating illumination geometries to be used in unison with high-performance refractive objective lenses and a visible light source. The PI signal originates from a nonradiative relaxation process after exciting a molecular vibration from the ground state through the absorption of a resonant IR photon. The absorbed energy stimulates nuclear motion that is then dispersed through phonons to the surrounding environment, causing a local temperature increase of 2 to 3 K and a corresponding thermally induced change to the refractive index. This change to the sample’s optophysical properties lasts 1 to 3  μs and is detectable by various means, including optical microscopy, photoacoustic, and atomic force microscopy based measurements. Conceptually, this is the same as SRP contrast except that the excited vibrational state is populated through an IR transition. Despite the long wavelength used to resonantly excite vibrational motion, once the IR photon is absorbed and the relaxation occurs, the induced temperature gradient remains in the local milieu and can be probed at much higher resolution using a diffraction-limited visible probe source. In PI microscopy, an image is captured with the sample under interrogation being simultaneously illuminated by pulsed visible and MIR light sources (the hot frame), followed by an image in which the MIR source is blocked (the cold frame)—the difference between the two images is the PI signal.

### 3.3 Phase Imaging Integration

PI microscopy has been demonstrated in point-scanning and widefield geometries for 2D and 3D imaging applications with ∼10  μM chemical concentration sensitivity.^62^ The effect however can be quite small (nanometer-scale thermal expansions and $∼10^{-4}$ changes to the refractive index).^63^^,^^64^ Observing nanometer-scale changes through intensity-based measurements, e.g., confocal or darkfield, is challenging, requiring many frame averages, which increases light dosage and limits throughput. For this reason, researchers are moving toward phase-based techniques that have sensitivity to optical path length (OPL) at the nanometer scale to achieve greater modulation depths between the hot and cold frames.^65^^,^^66^ Quantitative phase imaging (QPI) has emerged as a valuable method for investigating unlabeled specimens by providing quantitative maps of OPL delays introduced by the sample with a lower LOD for chemical concentrations that are inaccessible via intensity-based PI measurements.^67^ Combining the sensitivity of QPI with the chemical specificity of PI microscopy is potentially transformative. Although there are many variations of QPI, the most powerful approaches benefit from a common-path geometry for mechanical stability, single-frame acquisition for high-throughput, and white-light illumination from, for example, lamps or light emitting diodes (LEDs) to eliminate speckle noise and improve resolution.

Two of the most sensitive QPI techniques, invented by G. Popescu, are spatial light interference microscopy (SLIM)^68^^,^^69^ and gradient light interference microscopy (GLIM),^70^^,^^71^ which are the quantitative counterparts to Zernike phase-contrast and differential interference contrast microscopy, respectively. GLIM exhibits strong optical sectioning and is suitable for capturing 3D tomographic images, and SLIM can produce topographical images, which is useful for rapidly probing the total metabolite content within cells. SLIM and GLIM represent two complementary common-path interferometer geometries that use partial coherence white-light illumination for speckle-free phase reconstruction with sub-nanometer path-length stability. As on-axis interferometers, they leverage temporal phase-shifting in which, typically, four phase-shifted images are captured in π/2 increments. This preserves the high-resolution space-bandwidth product but at the expense of the time-bandwidth product. Although offering superior imaging quality, SLIM and GLIM modalities are less desirable for rapid cell screening given that multiple raw intensity images are necessary to produce a single-phase image. This detriment is further exaggerated considering that PI contrast requires both hot and cold frames, totaling eight frames needed to produce a single PI–QPI image, i.e., reducing the framerate of the imaging sensor by 8×.

On the other hand, off-axis, common-path interferometric methods such as diffraction phase microscopy (DPM)^72^ enable rapid QPI from a single frame. However, DPM systems using laser-based illumination are plagued by spatial noise due to speckles and multiple reflections, providing limited space-bandwidth utilization of the imaging system but with the advantage of capturing quantitative phase information rapidly. Demonstrations have shown the possibility of single-shot computational SLIM,^73^ which uses deep learning to produce SLIM-quality phase maps from single-frame DPM images to remove speckle. Another compelling approach is white-light DPM),^74^ which is an off-axis method that uses plane wave white-light illumination and hence reduces speckle noise. These approaches hold promise as future directions for integrating QPI with PI for rapid, high-sensitivity, high-resolution molecular imaging.

**Fig. 4:**
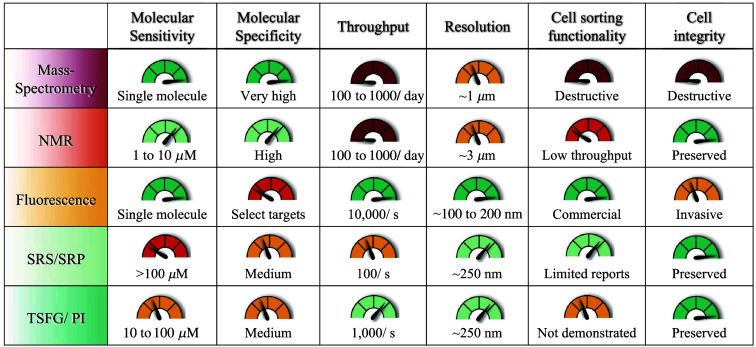
Comparison of analytical techniques used to characterize metabolite content for cell strain development efforts.

Despite the advantages of IR absorption as a potent method for deriving molecular contrast and the recent progress made in IR and phase microscopy, integrating IR imaging with microfluidics for rapid cell screening remains an underdeveloped field. This barrier originates from the strong IR absorption of many materials, including both polymers and glass, typically used to make microfluidic devices. Such materials are optically transparent in the visible and near-IR, making them suitable for fluorescence and Raman measurements, but they are opaque in the MIR. MIR-compatible microfluidic devices have been previously reported; they used standard microfabrication processes including etching and photolithography on IR transparent substrates including calcium fluoride (${\mathrm{CaF}}_{2}$) and barium fluoride (${\mathrm{BaF}}_{2}$).^75^^–^^77^ These fully sealed microfluidic devices with ∼50  μm channels offer the potential for rapid metabolite screening of living cells comparable to RIACS. The fact that SRS produces acceptable images of cells moving underflow in real time is an encouraging prelude to using IR-based techniques. Unlike direct absorption measurements in IR microscopy, the background caused by non-resonant water absorption in the fluidic channel is minimal in the TSFG and PI modalities. In both cases, the signal has a strong dependency on confinement to the focal plane. When, e.g., hydroxyl bending modes in water overlap with the vibrational modes of interest, deuterated water can be used to circumvent this difficulty. With anticipated developments of IR-compatible fluidic devices equipped with multiple, parallel fluidic channels and active cell sorting actuators within reach, high-throughput cell screening and sorting based on label-free IR absorption contrast in a widefield format are a realistic possibility. Combining RIACS with IR-microfluidics, in particular, will allow both Raman-active and IR-active molecules to be targets for active cell sorting. To further improve the breadth of addressable metabolite targets with specificity, especially those with overlapping spectral features, the use of molecular probes for functional multiplexing is required (Fig. 4).

## 4 Functional Multiplexing

Much like MS and NMR, access to improved chemical specificity through Raman and IR vibrational spectro-microscopy has come online using stable isotope probes to resolve overlapping spectroscopic peaks and track specific biomolecules and metabolic pathways. The use of deuterium labeling has enabled scientists to track lipogenesis from glucose and protein metabolism and shows promise as a marker with little to no biological effect.^78^ Using heavier isotopologues, vibrational resonances are red-shifted; in the case of deuterated C–D moieties, the stretching mode is shifted to $∼2100\;{\mathrm{cm}}^{-1}$ and into the cellular “silent region” (∼1900 to $2600\;{\mathrm{cm}}^{-1}$), a region that is usually spectrally silent in biological systems, thus allowing for the detection of such vibrational probes with a high SNR and specificity. In addition to isotope labels, molecular tags have also been used in live cell imaging.^79^ In this category, labels are selected for their large cross-sections, which facilitates their detection in Raman or IR microscopy. Unlike bulky fluorescent molecules, molecular tags such as alkynes (C≡C), nitriles (–C≡N), and isonitriles (–N≡C) are minimally perturbative, reside in the silent region for high contrast, eliminate concerns regarding photobleaching, and are generally orthogonal due to their narrow resonant linewidths $∼10\;{\mathrm{cm}}^{-1}$ in vibrational spectroscopy.^80^ This potentially allows for very high biomarker multiplexing (>20), a feat inaccessible through fluorescence due to broad, overlapping spectral emissions. By attaching these moieties to biomolecules such as amino acids, choline, glucose, and nucleosides, it is possible to follow *de novo* synthesis of new compounds after cellular uptake. Moreover, biosynthesized pharmaceuticals that intrinsically contain these groups, such as erlotinib, can be imaged directly, allowing for the examination of their production and localization.^81^ Isotope and molecular probes can provide improved metabolite specificity and are likely to play a significant role in future cell strain development efforts.

## 5 Conclusion

In this article, we presented several of the most prominent techniques used to unveil the biochemical signatures of metabolites produced in microbes for biomanufacturing. The discussion provides researchers in cell strain development guidance on the benefits and challenges of the available analytical techniques. A natural direction of future research is to combine technologies to acquire multiparameter datasets and reveal a more complete and quantitative profile of the cellular metabolome. Using compressed sensing and ML algorithms to train datasets acquired from non-invasive, high-throughput screening modalities (e.g., RIACS and IR-microfluidics) on information-rich datasets acquired on destructive or slow instruments (MS and NMR) will likely prove fruitful and ultimately improve the rate of discovery for genetic and environmental conditions that lead to enhanced production of high-value metabolites. This approach may likewise find utility in the development of mammalian cell lines for biopharmaceutical manufacturing of biologics. The quest for scalable biomanufacturing necessitates technological advancements in the instrumentation used in biofoundries for cell screening. Once economically viable cell strains are realized, they will be used ubiquitously throughout the sustainable bioeconomy of the near future—the benefits of which are palpable.

## Data Availability

Data sharing is not applicable to this article as no new data were created or analyzed.
